# Genome-wide identification and expression analysis of the growth regulating factor (GRF) family in *Jatropha curcas*

**DOI:** 10.1371/journal.pone.0254711

**Published:** 2021-07-15

**Authors:** Yuehui Tang, Wei Cheng, Shen Li, Ying Li, Xiang Wang, Jiatong Xie, Yingying He, Yaoyu Wang, Yiru Niu, Xinxin Bao, Qian Wu

**Affiliations:** 1 College of Life Science and Agronomy, Zhoukou Normal University, Zhoukou, China; 2 School of Journalism and Communication, Zhoukou Normal University, Zhoukou, China; East Carolina University, UNITED STATES

## Abstract

*GRF* genes have been confirmed to have important regulatory functions in plant growth, development and response to abiotic stress. Although the genome of *Jatropha curcas* is sequenced, knowledge about the identification of the species’ *GRF* genes and their expression patterns is still lacking. In this study, we characterized the 10 *JcGRF* genes. A detailed investigation into the physic nut GRF gene family is performed, including analysis of the exon-intron structure, conserved domains, conserved motifs, phylogeny, chromosomal locations, potential small RNA targets and expression profiles under both normal growth and abiotic stress conditions. Phylogenetic analysis indicated that the 10 *JcGRF* genes were classified into five groups corresponding to group I, II, III, IV and V. The analysis of conserved domains showed that the motifs of *JcGRF* genes were highly conserved in *Jatropha curcas*. Expression analysis based on RNA-seq and qRT-PCR showed that almost all *JcGRF* genes had the highest expression in seeds, but very low expression was detected in the non-seed tissues tested, and four *JcGRF* genes responded to at least one abiotic stress at at least one treatment point. Our research will provide an important scientific basis for further research on the potential functions of *JcGRF* genes in *Jatropha curcas* growth and development, and response to abiotic stress, and will eventually provide candidate genes for the breeding of *Jatropha curcas*.

## Introduction

Growth-regulating factors (GRFs) are plant-specific transcription factors with important regulatory functions in plant development and adaptation to environmental stress, which are characterized by two highly conserved domains, namely QLQ and WRC [1]. QLQ domain is an important protein-protein interaction domain, whereas WRC domain is a kind of plant-specific motif, which is mainly responsible for the interaction between transcription factors and DNA [2]. Since the first GRF family gene, namely *OsGRF1*, is isolated and functionally studied in rice [3], many *GRF* genes have been identified and characterized in a variety of plant species such as tomato, soybean, rice, cotton, streptophyta and *Arabidopsis* [2, 4–7].

In recent years, extensive studies in *Arabidopsis* and other crops indicate that *GRF* genes have crucial roles in mediating seeds, roots, leaves and fibers development [7–9]. For example, rapeseed *BnGRF2a* gene acts in regulating leave size, seed weight, and oil content, and *BnGRF2a* transgenic *Arabidopsis* plants have larger leaves (20% increase in leaves size), higher seed mass and oil content, and even oil content increased by 50% [10]. In *Arabidopsis*, *AtGRF9* overexpression plants have significantly inhibited leaf growth [7], whereas the *arf3* mutant reduced the size of the leaves by approximately 15% compared to the wild type [8]. In rice, overexpression of *OsGRF1* causes various physiological defects in plants, including leaf curling, delayed flowering, and imperfect development of carpels [3]. *OsGRF4*, regulated by OsmiR396, increasing *OsGRF4* expression significantly increases rice grain length, grain width and grain weight, indicating that *OsGRF4* plays a positive regulator role in regulating grain size [11]. *OsGRF6* positively regulates auxin synthesis, promotes inflorescence development, and increases spike number [12]. In tomato, *GRF* genes regulate fruit development [13]. In addition to the functions described above, recent researches have reported the involvement of *GRF* genes in the regulation of plant responses to abiotic stress [1, 9]. For example, *AtGRF7* gene is reportedly involved in response to abiotic stress in *Arabidopsis* [14], and the *atgrf7* mutant shows higher resistance to salt and drought stress compared with wild-type plants [9]. In summary, the results further indicate that GRF transcription factors play a key role in regulating the balance of plant growth and stress response. Thus, although many studies have shown that the *GRF* genes play important regulatory role in controlling plant growth and development, and employed to cultivate high-yield and high-quality crop varieties, there is still very little information available in species of the Euphorbiaceae family, especially *Jatropha curcas*.

*Jatropha curcas* (physic nut) is a non-edible crop that is widely distributed in tropical and subtropical regions. In recent years, the *Jatropha curcas* has become a star tree species owing to its high oil content in seeds (about 58%), and the overall performance of seed oil as a biofuel is better than soybean and rapeseed oil [15]. Additionally, the completion of *Jatropha curcas* genome sequencing can offer a further opportunity to explore gene resources for *Jatropha curcas* molecular improvement [16]. However, the roles of GRF proteins in *Jatropha curcas* have not yet been explored, although they play key roles in the formation of plants’ organs and stress responses [1, 9].

In this study, we attempted to establish a more comprehensive picture of the GRF gene family in *Jatropha curcas*. Firstly, we identified the *JcGRF* genes of *Jatropha curcas* from genome database. Secondly, we characterized the exon-intron structure and conserved domains of these genes, then subjected them to phylogenetic analysis. Finally, we analyzed their expression profiles in different tissues at different developmental stages under non-stressed conditions, and in roots exposed to abiotic stress. Taken together, our results will not only provide a foundation for future functional investigation of the GRF family in *Jatropha curcas*, but will also provide a foundation for improving crops, especially *Jatropha curcas*, through genetic modification. Taken together, these data.

## Materials and methods

### Identification of *JcGRF* genes in *Jatropha curcas*

To identify *Jatropha curcas* GRF protein, HMM models of the WRC (PF08879) and QLQ (PF08880) domains of GRF proteins, were downloaded from PFam (http://pfam.sanger.ac.uk/). They were then used as query sequences in local HMM-based searches, setting E-values <0.01 [17]. In addition, to identify *Jatropha curcas* GRF proteins that might have been missed through HMM model searching, nine identified *Arabidopsis* GRF protein sequences were invoked as queries to perform a BLASTP search against the *Jatropha curcas* genome database. And then sequences were selected for further analysis if the E value was less than 1e^-10^. Next, the possible JcGRF proteins were confirmed by testing whether they contained two conserved domains (QLQ and WRC) using PFam and SMART [18] programs. Physical and chemical properties of the *JcGRF* genes, such as length of the coding region, number of amino acids, MW (molecular weight), pI (theoretical isoelectric point) of each *JcGRF* genes were analyzed using ExPASy ProtParam database (https://web.expasy.org/protparam/). Multiple sequence alignments of JcGRFs were performed using the DNAMAN 6.0 software.

### Gene structure and conserved motif analysis of *JcGRF* genes

The GSDS (Gene Structure Display Server) was used to analyze the exon-intron structure of all *JcGRF* genes by aligning coding sequences to their corresponding genomic DNA sequences. Conserved motifs were examined by submitting all full-length JcGRF protein sequences file to the MEME online program (http://meme-suite.org/tools/meme) with the following parameters: site distribution was set at zero or one occurrence per sequence, motif number was 15, and motif width should be between 8 and 100.

### Phylogenetic and chromosome location analysis of *JcGRF* genes

GRF protein sequences of *Populus trichocarpa*, *Ricinus communis* L. *Solanum lycopersicum*, *Lotus japonicus* and rice were downloaded from NCBI and PlantTFDB (http://planttfdb.cbi.pku.edu.cn/), *Jatropha curcas* sequences were downloaded from GenBank (http://www.ncbi.nlm.nih.gov/; available from DDBJ/EMBL/ GenBank under accession number AFEW00000000), and GRF protein sequences of *Arabidopsis* were acquired from TAIR (https://www.arabidopsis.org/). Complete alignment of the amino acid sequence was analyzed by ClustalX 1.83, and then phylogenetic trees were created by MEGA 10 software [19], using the neighbor-joining (NJ) method with 1000 bootstrap replications. Finally, iTOL (https://www.itol.org/) was used to draw and beautify phylogenetic trees [20]. Chromosomal locations of *JcGRF* genes were obtained as described by Wu [16], and linkage maps of the *JcGRF* genes were drawn with the MapChart software package.

### Small RNAs target prediction of *JcGRF* genes

The sense orientation of each target gene sequence was compared with the reverse complement of the *Jatropha curcas* miR396a sequences, and the potential target gene mRNAs that aligned closely with these miRNAs were identified.

### Analysis of cis-elements in the promoters of the *JcGRF* genes

The promoter sequences (2.0 kb upstream of the translation start site) of the *JcGRF* genes were identified by searching the *Jatropha curcas* genome database (DDBJ/EMBL/GenBank under accession no. AFEW00000000), and the cis-elements in the promoters were predicted using PlantCARE (http://bioinformatics.psb.ugent.be/webtools/plantcare/html/).

### Preparation of plant materials

Our study used *Jatropha curcas* (GZQX0401) as the wild type owing to its genome sequencing has been completed (DDBJ/EMBL/GenBank under accession no. AFEW00000000) [16]. GZQX0401 seeds provided by Wu’s research group [16], and came from the Key Laboratory of Plant Resources Conservation and Sustainable Utilization, South China Botanical Garden, Chinese Academy of Sciences, Guangzhou, China.

We used six-leaf stage *Jatropha curcas* roots, stem cortex and leaves, and seeds of 14, 19, 25, 29, 35, 41 and 45 days after pollination from the same plants for spatial and temporal expression pattern analysis. *Jatropha curcas* at six-leaf stage was watered with Hoagland solution containing 100 mM NaCl as a salt stress treatment according to previous reports [21, 22]. Similarly, the drought stress treatment was begun at the six-leaf stage (eight weeks after germination). For the drought treatment, irrigation was withheld. Roots were sampled after 2 h, 2 d, and 4 d of salt stress; after 2 d, 4 d, and 7 d of drought stress, and then were saved for further analysis.

### RNA isolation and qRT-PCR

The plant extraction kit from Megan was used to extract RNA from various tissues required for this study, whereas the first-strand cDNA synthesis kit from TAKARA was used to synthesize cDNA. We submitted the raw sequence data obtained through the standard protocols to the NCBI sequence read archive (the accession no. of drought stress data was PRJNA257901, whereas the salt stress was PRJNA244896). RNA-seq data processing and calculation methods were as follows. For annotation, all tags were mapped to the reference sequences including 500 bp genomic sequences behind the open reading frame, allowing no more than one nucleotide mismatch per tag. All the tags that mapped to reference sequences from multiple genes were filtered, and the remaining tags were considered to be unambiguous tags. For gene expression analysis, the number of expressed tags was calculated [23] and then normalized to TPM (number of transcripts per million tags).

qRT-PCR was operated in the LightCycler 480 quantitative PCR system, applying the following conditions: the temperature was kept at 95°C for 30 s, followed by 95°C for 5 s, then 60°C for 20 s, 72°C for 20 s, and the whole process contained 40 cycles. We used the 2^-ΔΔCT^ method to detect relative transcript abundance, and the *JcActin* gene was used as an internal control [21]. The primers used employed in S1 File.

### Statistical analysis

Three biological replicates were used for all experiments, and Duncan tests were used to assess the significance of differences in measured variables between the materials [24] with the SAS software package version 9. P< 0.05 was considered as statistical significance.

## Results

### Identification of *GRF* genes in *Jatropha curcas*

To identify GRF family members in *Jatropha curcas*, nine GRF proteins from *Arabidopsis* were used to as query sequences against the physic nut genome. In addition, the HMM *GRF* gene model was used to detect *JcGRF* genes that may have been missed. After predicting conserved QLQ and WRC domains, two candidate sequences (GenBank Accession JCGZ_12570 and JCGZ_24703) were removed because they contained only one of the conserved domains. Finally, 10 JcGRF proteins (we named JcGRF01 to JcGRF10), which contained both QLQ and WRC domains, were identified in *Jatropha curcas* and used for subsequent analysis. Physical and chemical characteristics of all identified 10 JcGRF proteins, including the length of the coding sequence (CDS), the number of amino acids, molecular weight (MW) and isoelectric point (pI), were analyzed and presented in Table 1. Among the 10 JcGRF, the amino acid sequences of these proteins ranged from 204 amino acid (aa) (JcGRF08) to 613 aa (JcGRF05), and the length of their corresponding CDS ranged from 615 bp to 1842 bp. Moreover, the pI values of JcGRF proteins were variable, ranging from 5.57 (JcGRF10) to 9.32 (JcGRF04), and their molecular weight was from 22.3 kDa (JcGRF08) to 66.4 kDa (JcGRF05).

**Table 1:** Summary of *JcGRF* genes encoding GRF proteins in *Jatropha curcas*.

Genes	Gene ID	Protein length (aa)	ORF length	pI	MW	Location	Domain
			(bp)		(kDa)		
*JcGRF01*	JCGZ_23645	419	1260	8.55	45.6	LG6	QLQ, WRC
*JcGRF02*	JCGZ_02569	340	1023	8.81	36.8	LG6	QLQ, WRC
*JcGRF03*	JCGZ_12568	332	999	8.71	36.1	LG7	QLQ, WRC
*JcGRF04*	JCGZ_14504	481	1446	9.32	52.2	LG8	QLQ, WRC
*JcGRF05*	JCGZ_15989	613	1842	6.95	66.4	LG8	QLQ, WRC
*JcGRF06*	JCGZ_15990	608	1827	8.07	66.3	LG8	QLQ, WRC
*JcGRF07*	JCGZ_20371	474	1425	7.85	50.9	LG9	QLQ, WRC
*JcGRF08*	JCGZ_20720	204	615	9.13	22.3	LG9	QLQ, WRC
*JcGRF09*	JCGZ_07476	398	1197	8.77	44.9	LG10	QLQ, WRC
*JcGRF10*	JCGZ_10610	396	1191	5.57	43.3		QLQ, WRC

### Conserved amino acid sequences within the QLQ and WRC domains

Studies have shown that GRF proteins are characterized by the presence of both WRC and QLQ conserved domains at the N-terminus [1]. Therefore, we further analyzed conserved characteristics of the QLQ and WRC domains of the deduced JcGRF proteins. Our results indicated that the JcGRF proteins contained the same characteristic regions named QLQ and WRC as do *Arabidopsis*, rice, poplar, and castor bean GRF proteins (S1 Fig). Results also showed that many amino acid residues distributed in the two domains were highly conserved among GRF proteins from four plant species, while amino acids were more conserved in the WRC domain than those in the QLQ domain (S1 Fig). For example, fourteen highly conserved amino acids (C_7_, R_9_, D_10_G_11_K_12_K_13_W_14_R_15_C_16_, K_26_Y_27_C_28_, H_31_, and R_38_) were included in the WRC domain, whereas only E_5_, Q_9_, and P_23_ were highly conserved in the QLQ domain.

### Phylogenetic analysis of GRF proteins

**Fig 1:**
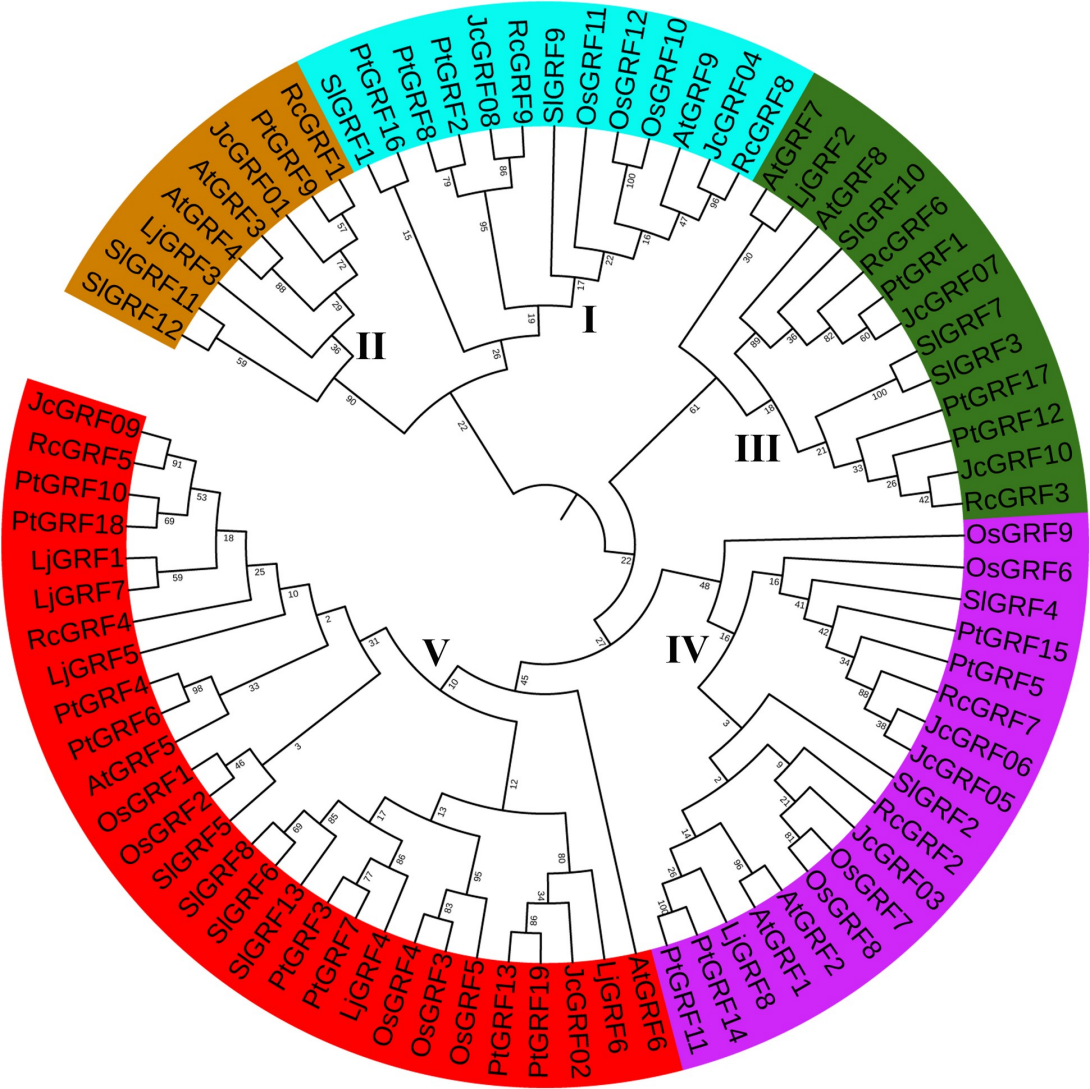
Phylogenetic tree of GRF protein. The NJ method (1000 bootstraps) was used to create the phylogenetic tree based on the similarity of full-length amino acid sequences of GRF proteins from physic nut, *Arabidopsis*, rice, castor bean, tomato, *Lotus japonicus* and poplar.

To gain more insights into the phylogenetic relationship between members of the GRF gene family, the full-length amino acid sequence of the GRF of the 80 proteins (S2 File) from 10 *Jatropha curcas*, 12 from rice, 9 from castor bean (*Ricinus communis* L.), 13 from tomato (*Solanum lycopersicum*), 8 from *Lotus japonicus*, 9 from *Arabidopsis* and 19 from poplar (*Populus trichocarpa*) were used to construct a phylogenetic tree. As shown in the resulting phylogenetic tree (Fig 1), the 80 GRF proteins were classified into five groups, designed group I (AtGRF9), II (AtGRF3/4), III (AtGRF7/8), IV (AtGRF5/6) and V (AtGRF1/2). Among the 10 identified JcGRF proteins from *Jatropha curcas*, group I contained JcGRF04 and 08, group II contained JcGRF01, group III contained JcGRF07 and 10, group IV contained JcGRF03, 05 and 06, and group V contained JcGRF02 and 09. Our results also showed that groups IV and V have a greater number of GRF proteins, and they had 14 and 19 members, respectively, and no rice GRF proteins were classified to groups II and III. Additionally, in the phylogenetic tree, some GRF proteins from *Arabidopsis*, rice and poplar formed related sister pairs, such as JcGRF05 and 06, PtGRF2 and 8, OsGRF1 and 2, OsGRF3 and 4, OsGRF7 and 8, OsGRF10 and 12, AtGRF3 and 4, PtGRF12 and 17, AtGRF1 and 2, PtGRF11 and 14, PtGRF4 and 6, PtGRF10 and 18, PtGRF13 and 19, PtGRF3 and 7, SlGRF3 and 7, SlGRF6 and 8, SlGRF11 and 12 (Fig 1). Taken together, these results suggested that GRF family members of different plants have undergone different evolutionary processes, and the JcGRF proteins showed a closer relationship with RcGRF proteins than with other GRF proteins.

In order to further confirm the reliability of the above classification results, another two trees were also constructed. Of the two phylogenetic trees, one from 10 *Jatropha curcas* GRF proteins (S2 Fig), and the other from 9 *Arabidopsis* GRF proteins and 10 *Jatropha curcas* GRF proteins (S3 Fig). The results indicated that these GRF proteins were divided into five groups (I-V), and the members of each group were the same as the above classification. These results further provide some support for the classification of GRF proteins from the above five plant species.

### Gene structure and conserved motif of *JcGRF* genes

**Fig 2:**
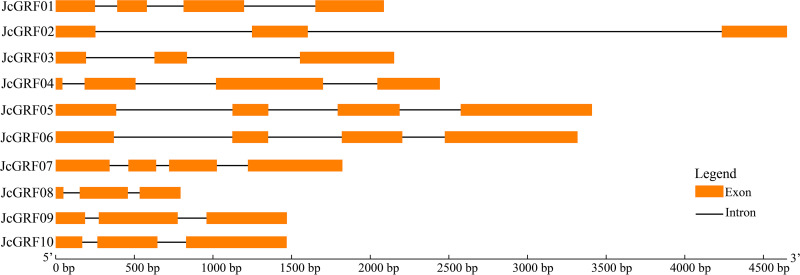
Gene structure in *JcGRF* genes from *Jatropha curcas*. Exons and introns are shown as light orange boxes and thin lines respectively.

To explore the exon-intron structure of *JcGRF* genes, the coding sequences and genomic sequences of these genes were aligned via the GSDS website. The results indicated that all of the coding sequences of the *JcGRF* genes were disrupted by different numbers of introns, and the numbers of introns of *JcGRF* genes varied between 2 and 3 (Fig 2). For example, *JcGRF1*, *5*, *6* and *7* contained three introns, while the remaining *JcGRF* genes contained two introns.

**Fig 3:**
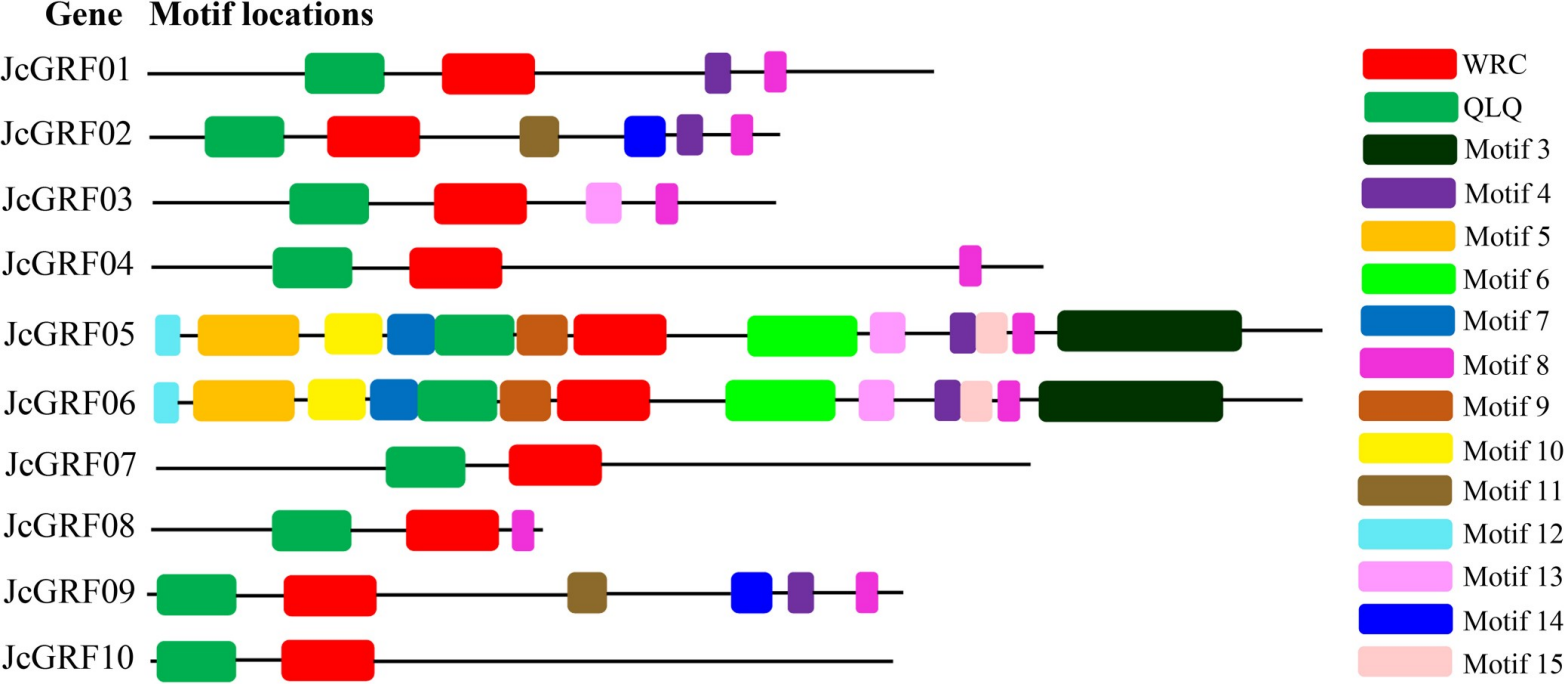
Conserved motifs of JcGRF protein obtained by MEME analysis. Different colored boxes represented different motifs, and were displayed on the right.

We further employed the MEME web server to examine the conserved motif in deduced JcGRF proteins, and fifteen motifs (named motif 1 to motif 15) were detected. As we predicted, motif 1 and motif 2, were characterized by WRC and QLQ domain, respectively, and were present in all JcGRF proteins (Fig 3 and S4 Fig). It was clear that JcGRF proteins within the same groups were usually found to share a similar conserved motif. For instance, motifs 11 and 14 existed only in group V, and motifs 3, 5, 6, 7, 9, 13, and 15 were only found in members of group IV. In short, the conserved motifs and similar exon-intron structures of the *GRF* genes in the same group, together with the phylogenetic analysis, strongly supported the phylogenetic relationships of GRF proteins.

### Chromosomal distribution of *JcGRF* genes

**Fig 4:**
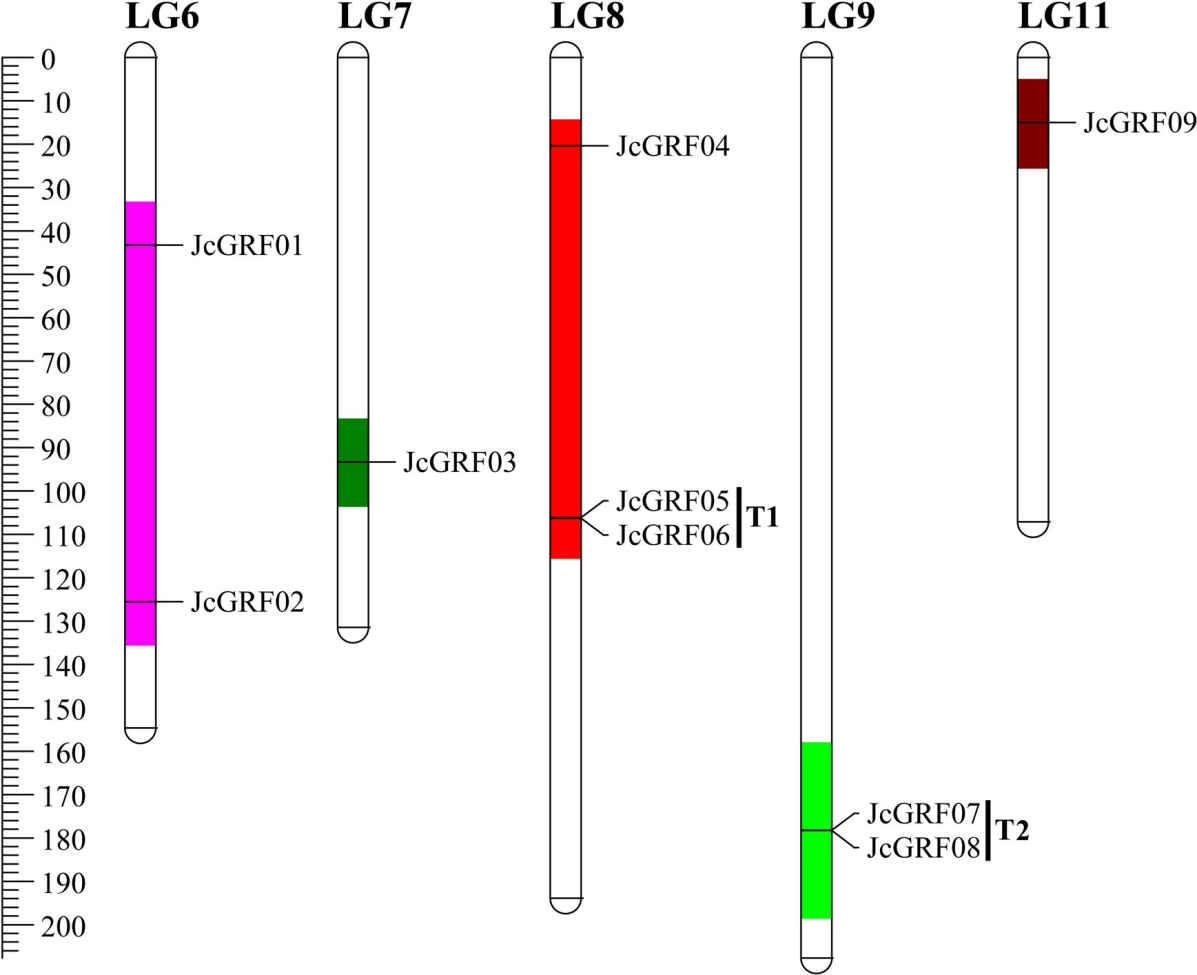
Location of *JcGRF* genes on *Jatropha curcas* chromosome. The number of chromosomes is displayed at the top.

The completion of the *Jatropha curcas* linkage groups (LGs) allows us to predict the location of the *JcGRF* genes on chromosome [16]. We observed that, excepted for *JcGRF10*, all JcGRF proteins found their location on the linkage groups (LGs) of *Jatropha curcas*. As shown in Fig 4, LG 8 had more members of the *JcGRF* gene family than other LGs, with three *JcGRF* genes. They were followed by LGs 6 and 9, each of which had two *JcGRF* genes, whereas each of LGs 7 and 11 had one *JcGRF* gene. In addition, no *JcGRF* genes were found in LGs 1, 2, 3, 4, 5 and 10. Previous studies have defined tandem duplications as tandem repeats of genes within 50 kb of each other or genes separated by <4 non-homologous spacers [25], also found in *Jatropha curcas JcGRF* genes. 40% (N = 4) of these *JcGRF* genes of *Jatropha curcas* exited as T (tandem repeats) at 2 loci on 2 LGs, and they were T1 (*JcGRF5* and *6*), and T2 (*JcGRF7* and *8*), located at LG 8 and LG9, respectively (Fig 4).

### Prediction of potential targets for small RNA

**Fig 5:**
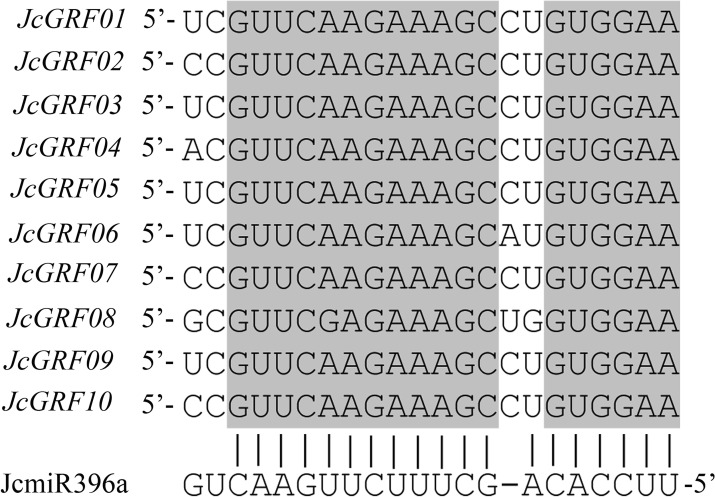
miR396a can target *JcGRF* genes. Diagram representing the *JcGRF* genes and miR396a. The interaction of 10 miR396a-regulated *JcGRFs* from *Jatropha curcas* with miR396a is indicated.

Researches show that most GRFs are target genes of microRNA396 (miR396), which participate in the growth and development of various plants along with miR396 [6, 8, 26, 27]. As the small RNA and its targets are highly conserved in various plant species, we tried to find potential small RNA targets for *JcGRF* genes by using the multiple sequence alignment and MiRanda software package. Our data suggested that all *JcGRF* genes contained very conserved fragments reverse complementary to miR396a (Fig 5), suggesting these members from the *Jatropha curcas* GRF gene family may be miR396a targets. Collectively, these results indicate that miR396a may be crucial for controlling the function of the *JcGRF* genes in regulating the growth and development of *Jatropha curcas* by decreasing their transcription.

### Prediction of cis-acting regulatory elements in the promoter region of *JcGRF* genes

The promoter regions 2000 bp upstream of 10 *JcGRF* genes were analyzed using PlantCARE, and this helped us better understand the potential functions of *GRF* genes in *Jatropha curcas*. As shown in S3 File, the cis-elements of *JcGRF* genes were classified into three major functional categories, plant growth and development, phytohormone responsive, and abiotic or biotic stress. In terms of plant growth and development, the CAT-box (cis-acting regulatory element related to meristem expression) was the largest of the 5 types of cis-acting regulatory element whose present was 7, whereas the HD-ZIp-1_motif was the smallest. As for plant phytohormone responsive, including ABRE (abscisic acid responsiveness), CGTCA-motif (MeJA-responsiveness), GARE-motif (gibberellin-responsive element), P-box (gibberellin-responsive element), TCA-element (salicylic acid responsiveness), TGA-element (auxin-responsive element) and AuxRR-core (auxin responsiveness) (S3 File). Moreover, low-temperature responsiveness (LTR), cold- and dehydration-responsiveness (C-repeat/DRE) and defense and stress responsiveness (TC-rich repeats) were presented in two, four and six *JcGRF* genes, respectively. Wound-responsive element (WUN motif) and MYB binding site involved in drought-inducibility appeared 7 and 7 times, accounting for 70% and 70% of the stress-related cis-acting elements, respectively. However, cis-acting elements related to anaerobic induction (ARE) and enhancer-like element involved in anoxic specific inducibility (GC motif) were only identified in nine and one genes, respectively.

### Expression profiles of *JcGRF* genes

**Fig 6:**
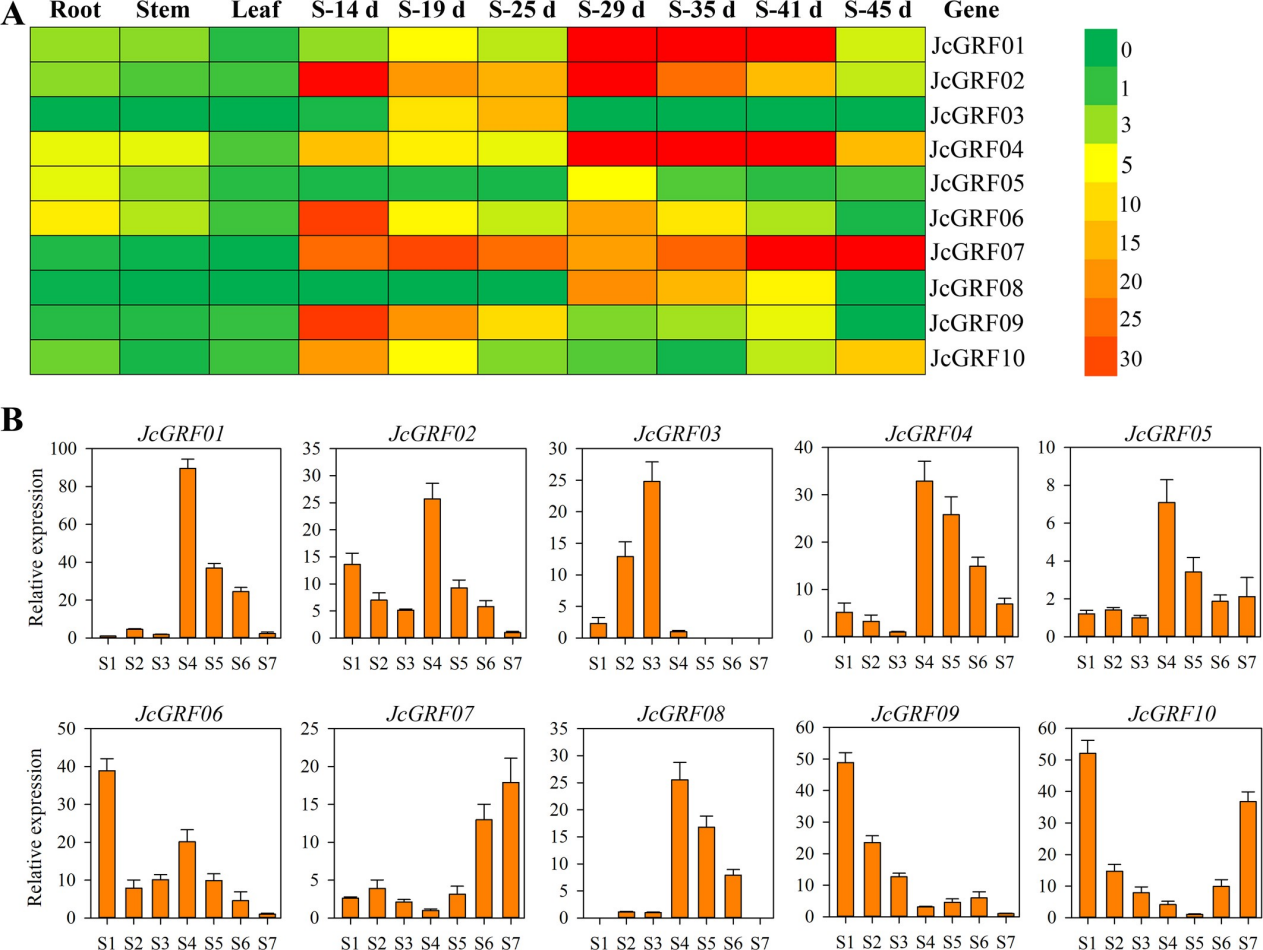
Transcription abundance of *JcGRF* genes in various tissues of *Jatropha curcas* based on RNA-seq and qRT-PCR data. (A) Patterns of expression of each *JcGRF* gene in *Jatropha curcas* roots, stem cortex, leaves, and seeds of 14 (S1), 19 (S2), 25 (S3), 29 (S4), 35 (S5), 41 (S6) and 45 (S7) based on RNA-seq, with a colored scale indicating expression levels shown on the right. NA means not available. (B) The expression of *JcGRF* genes in seeds of 14 (S1), 19 (S2), 25 (S3), 29 (S4), 35 (S5), 41 (S6) and 45 (S7) days after pollination was detected by qRT-PCR. Values represent means of n = 3 ± SD from three biological repeats.

To clarify the roles of *JcGRF* genes in regulating *Jatropha curcas* growth and development, we determined the expression profiles of these genes in roots, stem cortex, leaves, and seeds at different developmental stages (seeds of 14, 19, 25, 29, 35, 41 and 45 days after pollination) under non-stressed growth conditions based on data from RNA sequencing (RNA-seq) (Fig 6A and S4 File). Our results showed that almost all *JcGRF* genes (except *JcGRF06* expression in roots) were weakly expressed in roots, stem cortex and leaves (this value was less than five TPM), whereas these genes were highly expressed in seeds at least at one stage, with TPM values greater than five (Fig 6A). For example, *JcGRF03* and *JcGRF08*, no expression was detected in roots, stem cortex, and leaves, but the former was observed to be expressed during the early stages of seed development and the highest expression was found at 25 days after pollination, and the latter preferred to be expressed during the late stages of seed development especially in seeds for 29 days. Additionally, we found that *JcGRF07* and *JcGRF09* exhibited extremely weak expression in non-seed tissue tested (TPM<1), while they were most strongly expressed in seeds. In developing physic nut seeds, two genes (*JcGRF03* and *09*) preferred to be expressed in seeds of 14, 19 and 25 days compared to other stages, five genes (*JcGRF01*, *02*, *04*, *06* and *08*) continued to decrease in expression from 29 to 45 days, one gene (*JcGRF07*) showed continuous increase in expression from 29 to 45 days (Fig 6A). Taken together, members of the GRF family have the highest expression levels in seeds in *Jatropha curcas*, suggesting that these *JcGRF* genes may play important roles in the regulation of *Jatropha curcas* seed development.

In order to test the reliability of RNA-seq data, we further analyzed the transcription level of *JcGRF* genes at different developmental stages of seeds by qRT-PCR method (Fig 6B). The results suggested that the expression level of *JcGRF* genes detected by qRT-PCR technology was consistent with the RNA-seq data, indicating that the RNA-seq data was reliable and could lay a theoretical foundation for future research on the function of the *JcGRF* genes in regulating seed development.

### Expression profiles of *JcGRF* genes under drought and salinity stress

**Fig 7:**
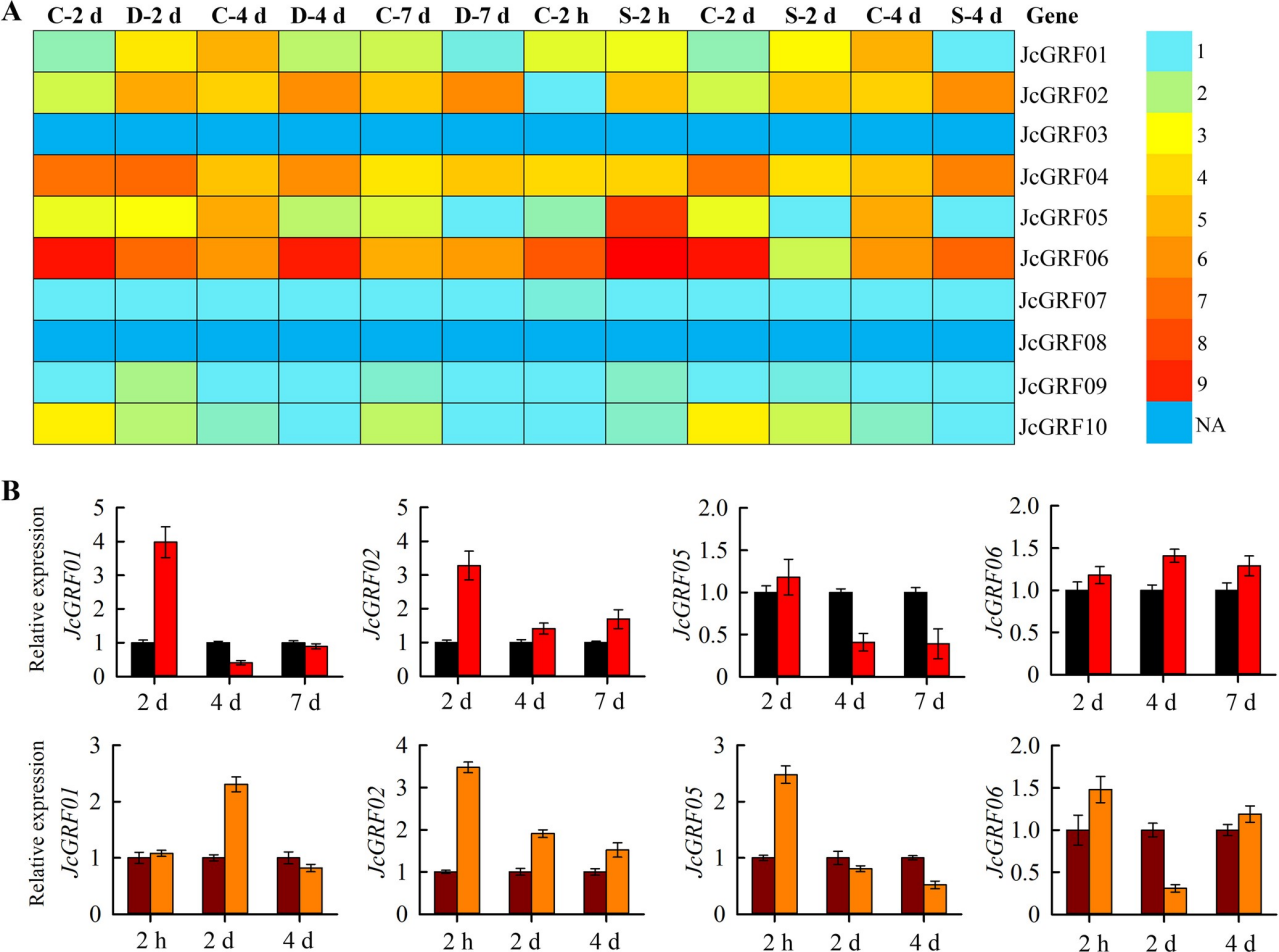
Transcription abundance of *JcGRF* genes in *Jatropha curcas* roots under drought and salinity stress. (A) The transcription level of *JcGRF* genes exposed to drought and salt stress, with a colored scale of expression levels shown on the right. NA (not available), C (Control), D (Drought), S (Salinity). (B) Relative expression levels of *JcGRF* genes in *Jatropha curcas* roots by qRT-PCR under drought (2 d, 4 d and 7 d) and salinity (2 h, 2 d and 4 d) stresses, and non-stressed treatment as control. Values represent means of n = 3 ± SD from three biological repeats.

We further analyzed the expression of *JcGRF* genes in roots under abiotic stress. As shown in Fig 7, most *JcGRF* genes had weak (TPM<5) or even no detectable expression in non-seed tissues tested under abiotic stress, and the effects of stress treatment on these genes were not obvious. However, very few genes showed differential expression compared to the control group. For instance, *JcGRF01*, *02* and *05* responded to drought and salt stress, whereas *JcGRF06* responded only to salt stress (Fig 7A).

To further confirm that some *JcGRF* genes responded to drought or salt stress, differentially expressed genes were selected for qRT-PCR analysis. The results showed that the *JcGRF* genes expression results detected by qRT-PCR had a similar change trend compared with the RNA-Seq results (Fig 7B), indicating that our expression profile data was reliable and could provide candidate genes for the cultivation of stress-tolerant varieties of *Jatropha curcas*.

## Discussion

Growth-regulating factors (GRFs) are a kind of plant-specific transcription factors, and one of the first-selected factors for crop genetic improvement, which play an important regulatory role in plant growth and development [6]. *Jatropha curcas*, as one of the most promising biomass energy crops, is rich in extremely high oil in its seeds [28]. However, there is litter knowledge of the identities, expression profiles of its *GRF* genes. Thus, we identified, characterized and detected the level of *GRF* genes expression in the species.

In our study, our identified 10 JcGRF proteins, and these proteins were divided into 5 groups (Fig 1). Phylogenetic tree showed that groups II and III contained GRF proteins from castor bean, *Arabidopsis*, *Jatropha curcas*, *Lotus japonicus*, tomato and poplar, whereas no rice GRF proteins were found, suggesting that the members of these groups may have been either remained in castor bean, *Arabidopsis*, *Jatropha curcas*, *Lotus japonicus*, tomato and poplar or disappeared in rice during the evolutionary process before their common ancestors separated. Motif analysis indicated that the assignment of the motifs of the *Jatropha curcas* GRF protein in different groups was highly variable, and conversely, there was a similar conserved motif among members of the same group (Fig 3), supporting the evolutionary conservation among GRF family members. Similar results also existed in various plants, such as *Arabidopsis*, grape, poplar, soybean, and rice [1, 13]. Collected, these results support the conservation of the evolution of the protein encoded by the *Jatropha curcas* GRF family gene, just as it does in other crops.

Studies have shown that *miR396* can directly down-regulate the transcription of *GRF* genes by degrading their mRNA [25, 29, 30]. For example, the expression of *AtGRF* genes in miR396a overexpressing plants is significantly lower than that in the control group [29]. In rice, overexpression of miR396 significantly reduced the expression of *OsGRF* genes [30]. Similarly, our results also suggested that these members from the *Jatropha curcas* GRF gene family may be miR396a targets (Fig 5). Collectively, the function of miR396 to inhibit *JcGRF* genes expression is also highly conserved in *Jatropha curcas*, as in *Arabidopsis* and rice.

Gene expression patterns can help researchers to further explore the biological characteristics of plant species (stress resistance, developmental regulation and tissue specificity), and lay the foundation for subsequent functional research [31, 32]. We, therefore, detected the abundance of *JcGRF* genes transcription based on these data from RNA-seq and qRT-PCR results. Previous studies have shown that *OsGRF4* regulates seed size by promoting cell division and cell expansion [11], the expression of its homologous genes *JcGRF02* and *JcGRF09* were significantly higher in seeds than in non-seeds in *Jatropha curcas* (Fig 6), showing that *JcGRF02* and *JcGRF09* might be related to *Jatropha curcas* yield traits, to be further studied. *OsGRF6* participates in regulating the rice number of grains/spike [12], and the gene with the highest homology level, *JcGRF03*, have high expression in seeds, showing that its function may be related to the growth and development of seed. Transgenic rice plants overexpressing *OsGRF10* exhibit fewer tillers [33], and its highest homologous gene *JcGRF04* may have an important function in regulating plant tillering. *Arabidopsis AtGRF1*, a target of miR396, functions as a development regulator in root cell reprogramming [26]. Similarly, our research also detected a miR396a target site on the mRNA sequence of its homologous gene *JcGRF05* (Fig 5), and *JcGRF05* had the highest transcription abundance in the root. These results suggested that *JcGRF05* might have important regulatory functions in *Jatropha curcas* root development, and mediated by miR396a, which may regulate a decrease in the level of *JcGRF05* gene expression. The *Jatropha curcas JcGRF06* gene was highly expressed in seeds (Fig 6), and its homologous gene *BnGRF2a* in rapeseed can significantly increase the weight and oil content of transgenic plant seeds [10], indicating that *JcGRF06* may be used for molecular improvement of *Jatropha curcas* seed yield and oil content. It is worth mentioning that almost all *JcGRF* genes had higher transcriptional abundance in seeds (Fig 6), while previous reports have suggested that *GRF* genes prefer to be expressed in young non-seed tissue, such as *Arabidopsis GRF*, rice *GRF* [2, 7]. These data indicate that the *GRF* genes may perform different functions in different species. Based on the transcription levels of *JcGRF* genes in different tissues and previous research results, we speculate that *JcGRF* genes may have functions in seed yield, oil content and development in *Jatropha curcas*, and needs further research.

Numerous studies have shown that the *GRF* genes respond to drought or salt stress, and increasing or decreasing the expression of these genes can improve crop response to adversity stress [1, 14]. For instance, compared with wild-type plants, the *atgrf7* mutant shows higher resistance to salt and drought stress [14]. Although some studies have screened the *GRF* gene as a key component to participate in abiotic stress responses, we still lack complete information about whether members of the *Jatropha curcas* GRF family also respond to drought or salt stress. In our research, qRT-PCR analysis, together with RNA-seq data in response to drought and salt stress, enabled us to screen *JcGRF* genes that respond to drought or salt stress (Fig 7). For example, expression of *JcGRF01*, *02* and *05* was up-regulated or down-regulated by drought and salt stresses at one or more time points, whereas *JcGRF06* responded only to salt stress. Collectively, we preliminary judgment these *JcGRF* genes may play an important role in the response of *Jatropha curcas* to abiotic stress, and then applied to the cultivation of *Jatropha curcas* resistant varieties. Taken together, this study can provide new genetic resources for further explore the regulatory function of *JcGRF* genes in the development of *Jatropha curcas* and stress tolerance, especially with respect to their influences on seed development and yield.

## Conclusion

In our study, we identified 10 *JcGRF* genes in *Jatropha curcas*, and characterized their expression patterns under both normal growth and abiotic stress conditions. Phylogenetic tree analysis showed that GRF proteins were divided into five groups, and the structural similarities and conserved motifs of members of the same group further supported the classification of GRF proteins in *Jatropha curcas*. miR396a can target *JcGRF* genes. Expression levels analysis showed that *JcGRF* genes have high expression patterns in seeds, indicating that these genes may play important regulatory roles in seed development and yield traits in *Jatropha curcas*. Taken together, these data can provide valuable resources for further explore the potential function of GRF family members in *Jatropha curcas*.

## Supporting information

S1 Fig GRF protein conserved domain. (A) Schematic diagram of the conserved QLQ and WRC domains of GRF protein. (B) The sequence logos showed the highly conserved QLQ domain in the GRF proteins of Jatropha curcas, Arabidopsis and rice, respectively. (C) The sequence logos showed the highly conserved WRC domains in the GRF proteins of Jatropha curcas, Arabidopsis and rice, respectively.
(JPG)

S2 Fig Unrooted phylogenetic tree of the proteins of GRF family in *Jatropha curcas*. The amino acid sequences were aligned using ClustalW and the phylogenetic tree was constructed using the neighbor-joining method. Bootstrap values were calculated for 1000 replicates.
(TIF)

S3 Fig Unrooted phylogenetic tree of the proteins of GRF family in *Arabidopsis* and *Jatropha curcas*. The amino acid sequences were aligned using ClustalW and the phylogenetic tree was constructed using the neighbor-joining method. Bootstrap values were calculated for 1000 replicates.
(TIF)

S4 Fig Summary of *JcGRF* genes encoding GRF proteins in *Jatropha curcas*. The amino acid sequence of each conserved motif within each JcGRF protein is shown by a colored box.
(TIF)

S1 File Primers used in this study. (XLSX)

S2 File Sequences of proteins encoded by *GRF* genes in *Arabidopsis*, rice, castor bean, tomato, *Lotus japonicus* and poplar. (TXT)

S3 File Cis-acting element analysis of *JcGRF* genes. (XLSX)

S4 File Signal strength values for the expression of 10 *JcGRF* genes in tested tissues (root, stem cortex, leaf, and seed) based on RNA-seq. (XLSX)
