# Supplementary figures and images for: Genome-wide identification and expression analysis of the growth regulating factor (GRF) family in Jatropha curcas
Source: PLoS One. 2021 Jul 15;16(7):e0254711. doi: 10.1371/journal.pone.0254711 (PMC8282010; doi:10.1371/journal.pone.0254711)

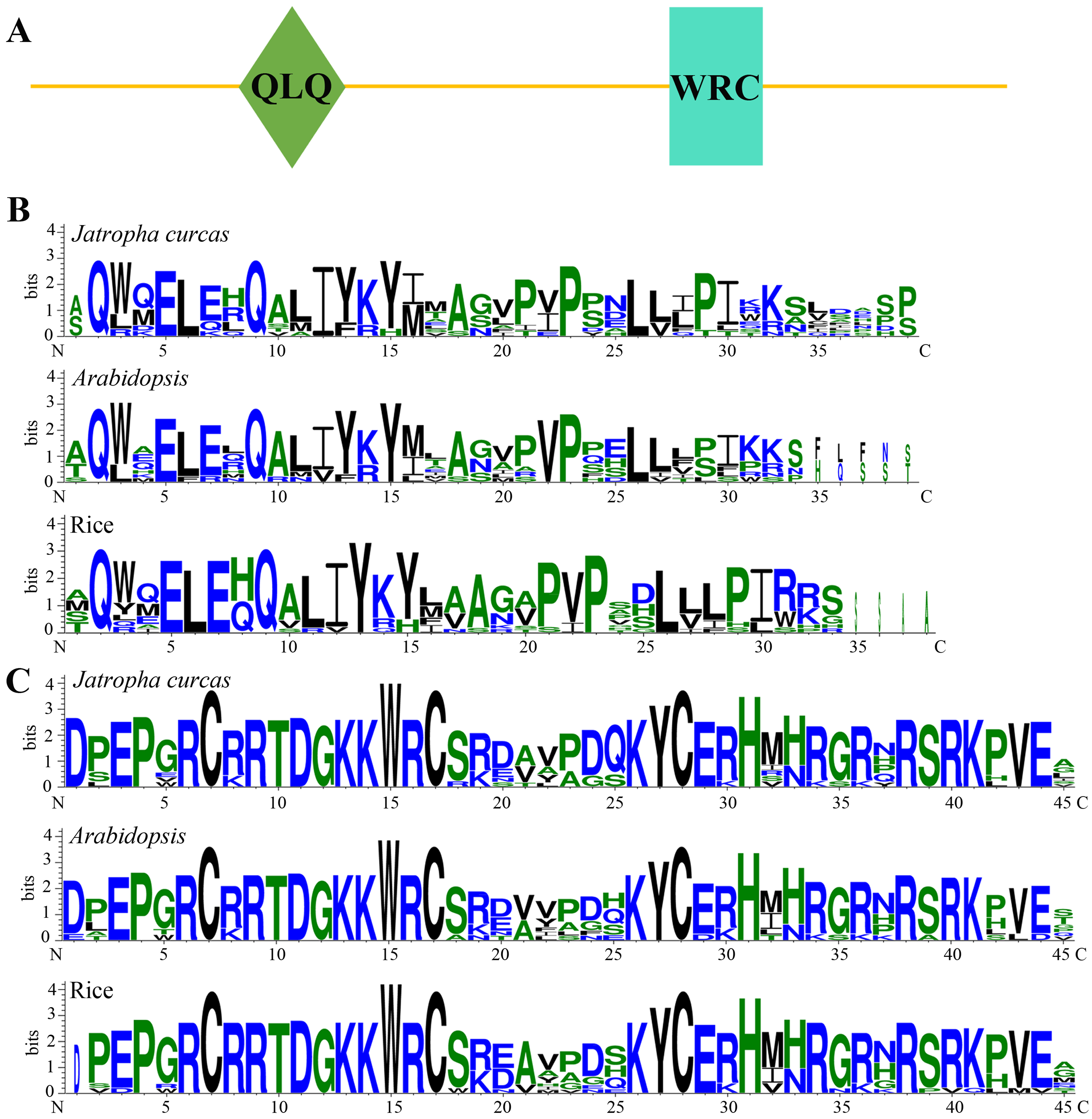

Supplement: S1 Fig — (A) Schematic diagram of the conserved QLQ and WRC domains of GRF protein. (B) The sequence logos showed the highly conserved QLQ domain in the GRF proteins of Jatropha curcas, Arabidopsis and rice, respectively. (C) The sequence logos showed the highly conserved WRC domains in the GRF proteins of Jatropha curcas, Arabidopsis and rice, respectively. (JPG) [file pone.0254711.s001.jpg]

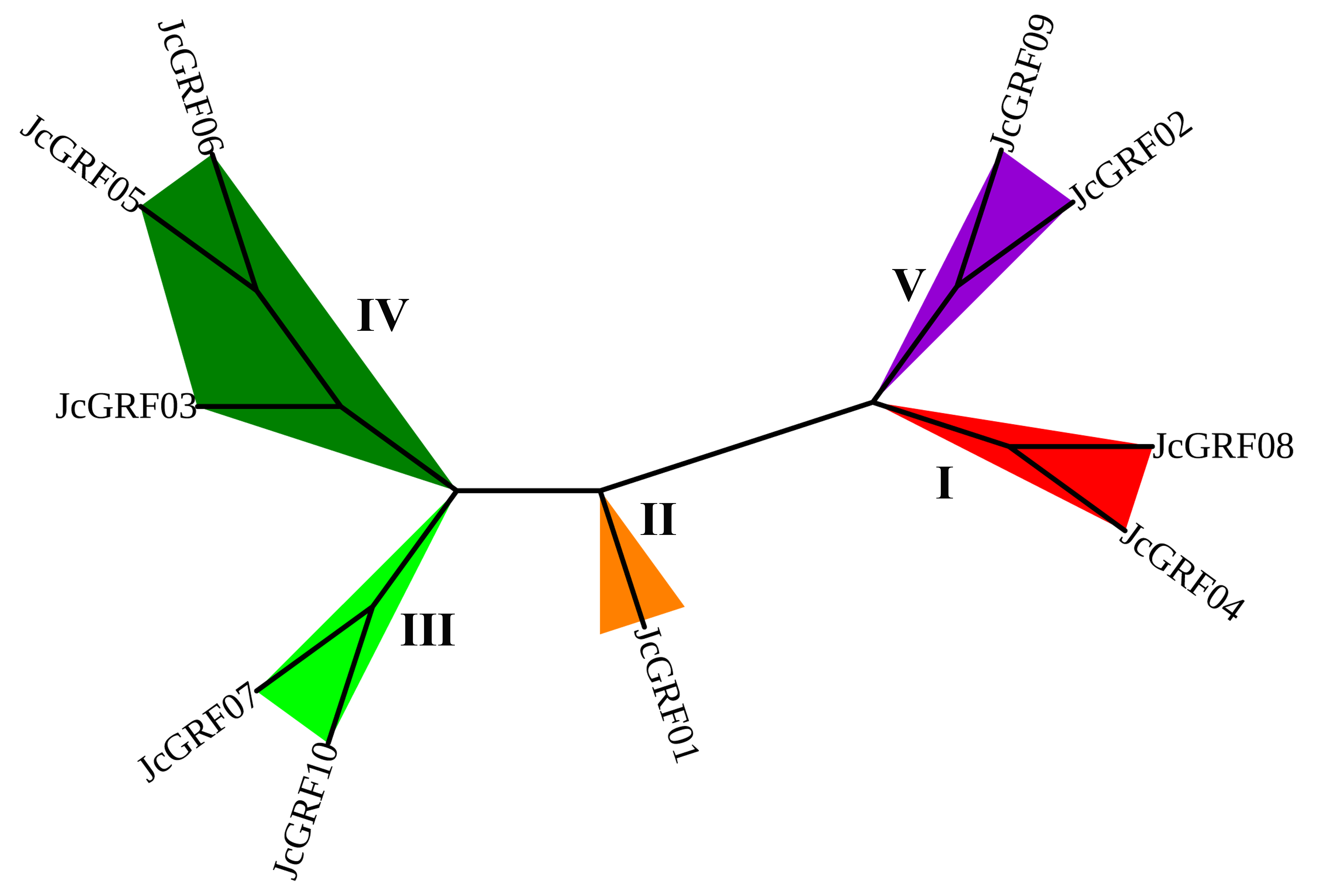

Supplement: S2 Fig — The amino acid sequences were aligned using ClustalW and the phylogenetic tree was constructed using the neighbor-joining method. Bootstrap values were calculated for 1000 replicates. (TIF) [file pone.0254711.s002.tif]

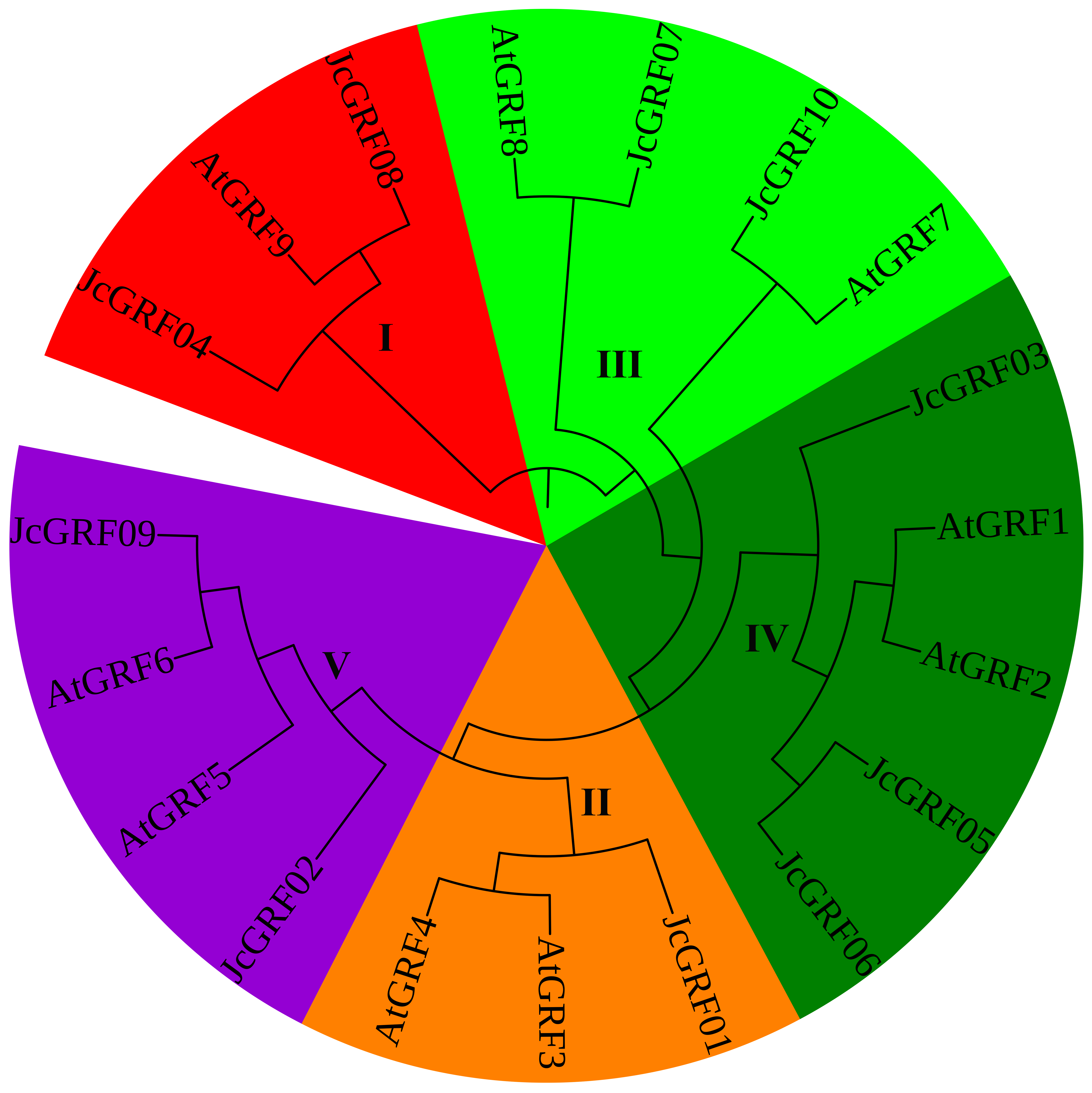

Supplement: S3 Fig — The amino acid sequences were aligned using ClustalW and the phylogenetic tree was constructed using the neighbor-joining method. Bootstrap values were calculated for 1000 replicates. (TIF) [file pone.0254711.s003.tif]

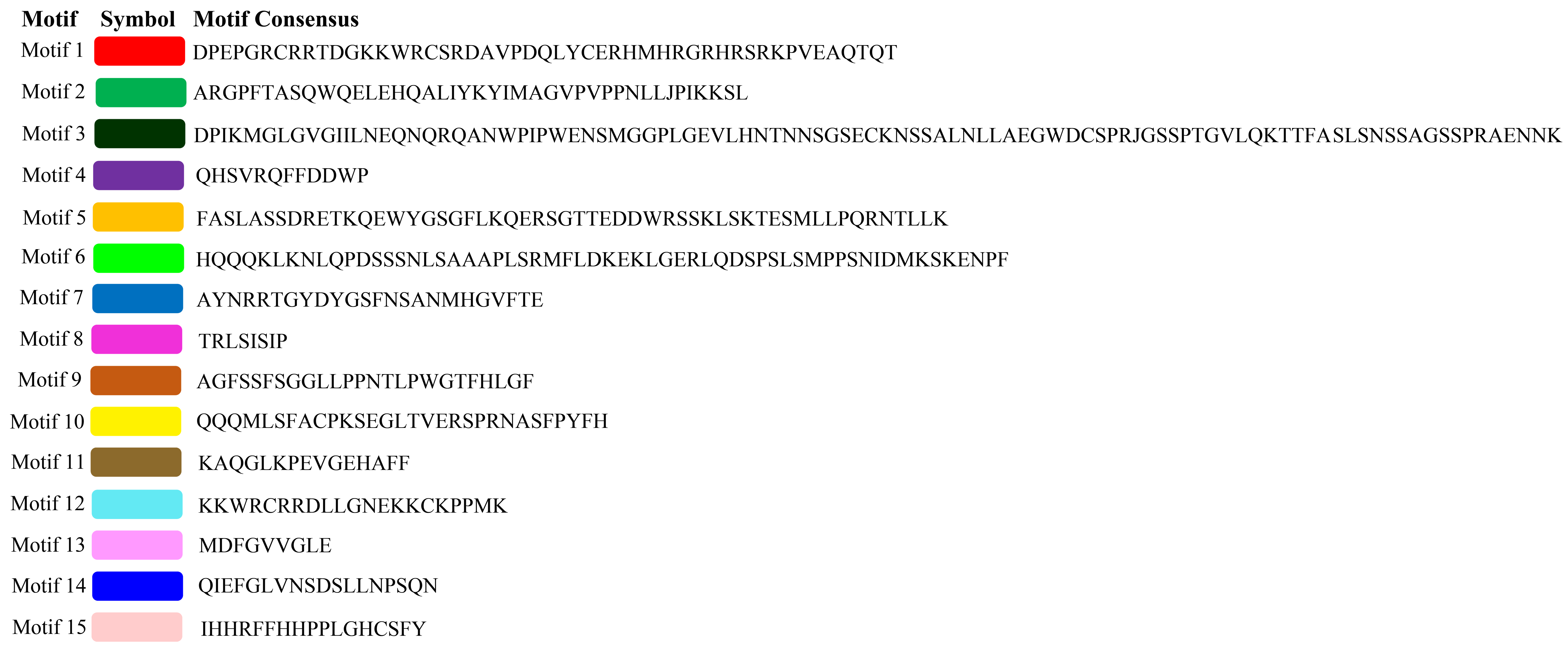

Supplement: S4 Fig — The amino acid sequence of each conserved motif within each JcGRF protein is shown by a colored box. (TIF) [file pone.0254711.s004.tif]
