# Supplementary material for: Genome-wide identification and expression analysis of the growth regulating factor (GRF) family in Jatropha curcas
Source: PLoS One. 2021 Jul 15;16(7):e0254711. doi: 10.1371/journal.pone.0254711 (PMC8282010; doi:10.1371/journal.pone.0254711)
Supplement: Attachment — Submitted filename: Respone to editor and Reviewers.docx [file pone.0254711.s009.docx]

Dear editor and reviewers:

We thank you very much for giving us an opportunity to revise our manuscript. Furthermore, thank you for your comments and suggestions concerning our manuscript entitled “Genome-wide identification and expression analysis of the growth regulating factor (GRF) family in *Jatropha curcas*”. The comments have all been very helpful to us in revising and improving our paper, as well as being of value in guiding our research. We have studied the comments carefully and have made corrections which we hope will meet with your approval. Revised portions of the paper are marked in red. In addition, the paper has been edited by a native-English speaker with a PhD in a relevant discipline working for the professional editing company Sees-editing Ltd.

We hope you will consider this work for publication in PLos One. Thank you again for your consideration.

**Comments to the Author**

Reviewer #1:

1. Figure 3: It is recommended that the authors rename motif 1 and 2 to WRC and QLQ to be more intuitive.

**Response:** Firstly, special thanks to you for giving me the opportunity to modify our paper. Secondly, thank you very much for your suggestion and your good comments. We have rename motif 1 and 2 to WRC and QLQ in the revised MS.

2. Figure 7B: It is recommended to indicate the treatment represented by each color.

**Response:** Special thanks to you for your good comments. We have added the treatment represented by different color in the revised MS, in the Figure 7B.

3. 154-155 line: You used two strategies instead of only blastP search.

**Response:** We appreciate for Reviewers’ suggestion. This is an important point that we should have mentioned. According to your advice, we have added two strategies instead of only blastP search in lines 160-161, the revised MS.

4. Figure 4: Why is each chromosome labeled in a different color and with a different range? Is there any special meaning?

**Response:** We appreciate for Reviewers’ comments. We use a different length color mainly for the purpose of making it easier for readers to find the location of the JcGRF proteins on the chromosome, and on the other hand for aesthetics.

5. Figure 1: The author needs to be in the analysis of evolution bootstraps values marked in the Figure, high bootstraps values on the branch gene deserves more attention, at the same time can also be found JcGRF genetic relationship with the distance of GRF gene in different species.

**Response:** Special thanks to you for your good comments. According to your advice, we have added the bootstraps values on the branch in the phylogenetic tree in Figure 1, as shown in the Revised MS.

6. The author needs to explicitly refer to the source of the software used, such as Line 110, MEGA 11 software.

**Response:** We appreciate for Reviewers’ suggestion. We have added references about MEGA X. We hope you will consider this work for publication in PLos One. Thank you again for your consideration.

7. 91-92 line: It is suggested that the authors submit the protein sequences of candidate genes to the NCBI CDD (https://www.ncbi.nlm.nih.gov/Structure/cdd/wrpsb.cgi) and SMART (http://smart.embl-heidelberg.de/smart/set_mode.cgi?NORMAL=1) databases for the presence of the conserved domain of GRF.

**Response:** We appreciate for Reviewers’ suggestion. Thank you for your suggestion. All GRF family candidate genes of *Jatropha curcas* have been uploaded to the NCBI database, and the gene numbers are shown in Table 1, in the revised MS.

8. It is recommended to add analysis experiments to make the article more complete, such as Gene Ontology annotation, Cis-acting elements analysis.

**Response:** Thank you very much for your good suggestion. According to your advice, we have added data on the analysis of cis-acting elements of the GRF gene, in lines 119-123, and 240-256, in the revised MS, as shown in S3 File.

9. It is better to put a separate paragraph on the brief introduction of content of this study (lines 75-82) and it should briefly introduce the significance and value of this research.

**Response:** We appreciate for Reviewers’ comments. We have made the modification according to your request. We hope you will consider this work for publication in PLos One. Thank you again for your consideration.
