# Supplementary material for: Genome-wide identification and expression analysis of the growth regulating factor (GRF) family in Jatropha curcas
Source: PLoS One. 2021 Jul 15;16(7):e0254711. doi: 10.1371/journal.pone.0254711 (PMC8282010; doi:10.1371/journal.pone.0254711)
Supplement: Peer review history

# Peer review: Decision Letter 0

**DOI:** 10.1371/journal.pone.0254711.r001

**Authors:** Baohong Zhang

21 Apr 2021

PONE-D-21-09573

Genome-wide identification and expression analysis of the growth regulating factor (GRF) family in Jatropha curcas

PLOS ONE

Dear Dr. Tang,

Thank you for submitting your manuscript to PLOS ONE. After careful consideration, we feel that it has merit but does not fully meet PLOS ONE’s publication criteria as it currently stands. Therefore, we invite you to submit a revised version of the manuscript that addresses the points raised during the review process.

Please submit your revised manuscript by Jun 05 2021 11:59PM. If you will need more time than this to complete your revisions, please reply to this message or contact the journal office at plosone@plos.org. When you're ready to submit your revision, log on to https://www.editorialmanager.com/pone/ and select the 'Submissions Needing Revision' folder to locate your manuscript file.

Please include the following items when submitting your revised manuscript:

- A rebuttal letter that responds to each point raised by the academic editor and reviewer(s). You should upload this letter as a separate file labeled 'Response to Reviewers'.
- A marked-up copy of your manuscript that highlights changes made to the original version. You should upload this as a separate file labeled 'Revised Manuscript with Track Changes'.
- An unmarked version of your revised paper without tracked changes. You should upload this as a separate file labeled 'Manuscript'.

If you would like to make changes to your financial disclosure, please include your updated statement in your cover letter. Guidelines for resubmitting your figure files are available below the reviewer comments at the end of this letter.

If applicable, we recommend that you deposit your laboratory protocols in protocols.io to enhance the reproducibility of your results. Protocols.io assigns your protocol its own identifier (DOI) so that it can be cited independently in the future. For instructions see: http://journals.plos.org/plosone/s/submission-guidelines#loc-laboratory-protocols. Additionally, PLOS ONE offers an option for publishing peer-reviewed Lab Protocol articles, which describe protocols hosted on protocols.io. Read more information on sharing protocols at https://plos.org/protocols?utm_medium=editorial-email&utm_source=authorletters&utm_campaign=protocols.

We look forward to receiving your revised manuscript.

Kind regards,

Baohong Zhang, Ph.D

Academic Editor

PLOS ONE

Journal Requirements:

When submitting your revision, we need you to address these additional requirements.

1. Please ensure that your manuscript meets PLOS ONE's style requirements, including those for file naming. The PLOS ONE style templates can be found at

https://journals.plos.org/plosone/s/file?id=wjVg/PLOSOne_formatting_sample_main_body.pdf and

https://journals.plos.org/plosone/s/file?id=ba62/PLOSOne_formatting_sample_title_authors_affiliations.pdf

2. Thank you for stating the following in the Acknowledgments Section of your manuscript:

'We acknowledge funding from the Natural Science Foundation of Henan Province (202300410520), Key Scientific Research Projects of the Higher Education Institutions of Henan province (21A180028), and Zhoukou Normal University College Students Innovation and Entrepreneurship Training Program (No. 202010478036 and No. 202010478003).'

We note that you have provided funding information that is not currently declared in your Funding Statement. However, funding information should not appear in the Acknowledgments section or other areas of your manuscript. We will only publish funding information present in the Funding Statement section of the online submission form.

a. Please remove any funding-related text from the manuscript and let us know how you would like to update your Funding Statement. Currently, your Funding Statement reads as follows: 'No'

b. Please include your amended statements within your cover letter; we will change the online submission form on your behalf.

[Note: HTML markup is below. Please do not edit.]

Reviewers' comments:

Reviewer's Responses to Questions

**Comments to the Author**

1. Is the manuscript technically sound, and do the data support the conclusions?

The manuscript must describe a technically sound piece of scientific research with data that supports the conclusions. Experiments must have been conducted rigorously, with appropriate controls, replication, and sample sizes. The conclusions must be drawn appropriately based on the data presented. 

Reviewer #1: Yes

**********

2. Has the statistical analysis been performed appropriately and rigorously? 

Reviewer #1: Yes

**********

3. Have the authors made all data underlying the findings in their manuscript fully available?

The PLOS Data policy requires authors to make all data underlying the findings described in their manuscript fully available without restriction, with rare exception (please refer to the Data Availability Statement in the manuscript PDF file). The data should be provided as part of the manuscript or its supporting information, or deposited to a public repository. For example, in addition to summary statistics, the data points behind means, medians and variance measures should be available. If there are restrictions on publicly sharing data—e.g. participant privacy or use of data from a third party—those must be specified.

Reviewer #1: Yes

**********

4. Is the manuscript presented in an intelligible fashion and written in standard English?

PLOS ONE does not copyedit accepted manuscripts, so the language in submitted articles must be clear, correct, and unambiguous. Any typographical or grammatical errors should be corrected at revision, so please note any specific errors here.

Reviewer #1: Yes

**********

5. Review Comments to the Author

Please use the space provided to explain your answers to the questions above. You may also include additional comments for the author, including concerns about dual publication, research ethics, or publication ethics. (Please upload your review as an attachment if it exceeds 20,000 characters)

Reviewer #1: 1.Figure 3: It is recommended that the authors rename motif 1 and 2 to WRC and QLQ to be more intuitive.

2.Figure 7B: It is recommended to indicate the treatment represented by each color.

3.154-155 line: You used two strategies instead of only blastP search.

4.Figure 4: Why is each chromosome labeled in a different color and with a different range? Is there any special meaning?

5.Figure 1: The author needs to be in the analysis of evolution bootstraps values marked in the Figure, high bootstraps values on the branch gene deserves more attention, at the same time can also be found JcGRF genetic relationship with the distance of GRF gene in different species.

6.The author needs to explicitly refer to the source of the software used, such as Line 110, MEGA 11 software.

7.91-92 line: It is suggested that the authors submit the protein sequences of candidate genes to the NCBI CDD (https://www.ncbi.nlm.nih.gov/Structure/cdd/wrpsb.cgi) and SMART (http://smart.embl-heidelberg.de/smart/set_mode.cgi?NORMAL=1) databases for the presence of the conserved domain of GRF.

8.It is recommended to add analysis experiments to make the article more complete, such as Gene Ontology annotation, Cis-acting elements analysis.

9.It is better to put a separate paragraph on the brief introduction of content of this study (lines 75-82) and it should briefly introduce the significance and value of this research.

**********

6. PLOS authors have the option to publish the peer review history of their article (what does this mean?). If published, this will include your full peer review and any attached files.

If you choose “no”, your identity will remain anonymous but your review may still be made public.

**Do you want your identity to be public for this peer review?** For information about this choice, including consent withdrawal, please see our Privacy Policy.

Reviewer #1: **Yes: **Dongfang Ma

[NOTE: If reviewer comments were submitted as an attachment file, they will be attached to this email and accessible via the submission site. Please log into your account, locate the manuscript record, and check for the action link "View Attachments". If this link does not appear, there are no attachment files.]

While revising your submission, please upload your figure files to the Preflight Analysis and Conversion Engine (PACE) digital diagnostic tool, https://pacev2.apexcovantage.com/. PACE helps ensure that figures meet PLOS requirements. To use PACE, you must first register as a user. Registration is free. Then, login and navigate to the UPLOAD tab, where you will find detailed instructions on how to use the tool. If you encounter any issues or have any questions when using PACE, please email PLOS at figures@plos.org. Please note that Supporting Information files do not need this step.

# Author comment: Author response to Decision Letter 0

**DOI:** 10.1371/journal.pone.0254711.r002

16 Jun 2021

Dear editor and reviewers:

We thank you very much for giving us an opportunity to revise our manuscript. Furthermore, thank you for your comments and suggestions concerning our manuscript entitled “Genome-wide identification and expression analysis of the growth regulating factor (GRF) family in Jatropha curcas”. The comments have all been very helpful to us in revising and improving our paper, as well as being of value in guiding our research. We have studied the comments carefully and have made corrections which we hope will meet with your approval. Revised portions of the paper are marked in red. In addition, the paper has been edited by a native-English speaker with a PhD in a relevant discipline working for the professional editing company Sees-editing Ltd.

We hope you will consider this work for publication in PLos One. Thank you again for your consideration.

Comments to the Author

Reviewer #1:

1. Figure 3: It is recommended that the authors rename motif 1 and 2 to WRC and QLQ to be more intuitive.

Response: Firstly, special thanks to you for giving me the opportunity to modify our paper. Secondly, thank you very much for your suggestion and your good comments. We have rename motif 1 and 2 to WRC and QLQ in the revised MS. 

2. Figure 7B: It is recommended to indicate the treatment represented by each color.

Response: Special thanks to you for your good comments. We have added the treatment represented by different color in the revised MS, in the Figure 7B.

3. 154-155 line: You used two strategies instead of only blastP search.

Response: We appreciate for Reviewers’ suggestion. This is an important point that we should have mentioned. According to your advice, we have added two strategies instead of only blastP search in lines 160-161, the revised MS.

4. Figure 4: Why is each chromosome labeled in a different color and with a different range? Is there any special meaning?

Response: We appreciate for Reviewers’ comments. We use a different length color mainly for the purpose of making it easier for readers to find the location of the JcGRF proteins on the chromosome, and on the other hand for aesthetics.

5. Figure 1: The author needs to be in the analysis of evolution bootstraps values marked in the Figure, high bootstraps values on the branch gene deserves more attention, at the same time can also be found JcGRF genetic relationship with the distance of GRF gene in different species.

Response: Special thanks to you for your good comments. According to your advice, we have added the bootstraps values on the branch in the phylogenetic tree in Figure 1, as shown in the Revised MS.

6. The author needs to explicitly refer to the source of the software used, such as Line 110, MEGA 11 software. 

Response: We appreciate for Reviewers’ suggestion. We have added references about MEGA X. We hope you will consider this work for publication in PLos One. Thank you again for your consideration.

7. 91-92 line: It is suggested that the authors submit the protein sequences of candidate genes to the NCBI CDD (https://www.ncbi.nlm.nih.gov/Structure/cdd/wrpsb.cgi) and SMART (http://smart.embl-heidelberg.de/smart/set_mode.cgi?NORMAL=1) databases for the presence of the conserved domain of GRF. 

Response: We appreciate for Reviewers’ suggestion. Thank you for your suggestion. All GRF family candidate genes of Jatropha curcas have been uploaded to the NCBI database, and the gene numbers are shown in Table 1, in the revised MS. 

8. It is recommended to add analysis experiments to make the article more complete, such as Gene Ontology annotation, Cis-acting elements analysis.

Response: Thank you very much for your good suggestion. According to your advice, we have added data on the analysis of cis-acting elements of the GRF gene, in lines 119-123, and 240-256, in the revised MS, as shown in S3 File.

9. It is better to put a separate paragraph on the brief introduction of content of this study (lines 75-82) and it should briefly introduce the significance and value of this research.

Response: We appreciate for Reviewers’ comments. We have made the modification according to your request. We hope you will consider this work for publication in PLos One. Thank you again for your consideration.

Attachment Submitted filename: Respone to editor and Reviewers.docx Click here for additional data file.

# Editor report: Decision Letter 1

**DOI:** 10.1371/journal.pone.0254711.r003

**Authors:** Baohong Zhang

2 Jul 2021

Genome-wide identification and expression analysis of the growth regulating factor (GRF) family in Jatropha curcas

PONE-D-21-09573R1

Dear Dr. Tang,

We’re pleased to inform you that your manuscript has been judged scientifically suitable for publication and will be formally accepted for publication once it meets all outstanding technical requirements.

Within one week, you’ll receive an e-mail detailing the required amendments. When these have been addressed, you’ll receive a formal acceptance letter and your manuscript will be scheduled for publication.

An invoice for payment will follow shortly after the formal acceptance. To ensure an efficient process, please log into Editorial Manager at http://www.editorialmanager.com/pone/, click the 'Update My Information' link at the top of the page, and double check that your user information is up-to-date. If you have any billing related questions, please contact our Author Billing department directly at authorbilling@plos.org.

If your institution or institutions have a press office, please notify them about your upcoming paper to help maximize its impact. If they’ll be preparing press materials, please inform our press team as soon as possible -- no later than 48 hours after receiving the formal acceptance. Your manuscript will remain under strict press embargo until 2 pm Eastern Time on the date of publication. For more information, please contact onepress@plos.org.

Kind regards,

Baohong Zhang, Ph.D

Academic Editor

PLOS ONE

Additional Editor Comments (optional):

Reviewers' comments:

# Editor report: Acceptance letter

**DOI:** 10.1371/journal.pone.0254711.r004

**Authors:** Baohong Zhang

8 Jul 2021

PONE-D-21-09573R1 

Genome-wide identification and expression analysis of the growth regulating factor (GRF) family in *Jatropha curcas*

Dear Dr. Tang:

I'm pleased to inform you that your manuscript has been deemed suitable for publication in PLOS ONE. Congratulations! Your manuscript is now with our production department. 

If your institution or institutions have a press office, please let them know about your upcoming paper now to help maximize its impact. If they'll be preparing press materials, please inform our press team within the next 48 hours. Your manuscript will remain under strict press embargo until 2 pm Eastern Time on the date of publication. For more information please contact onepress@plos.org.

If we can help with anything else, please email us at plosone@plos.org. 

Thank you for submitting your work to PLOS ONE and supporting open access. 

Kind regards, 

PLOS ONE Editorial Office Staff

on behalf of

Professor Baohong Zhang 

Academic Editor

PLOS ONE
